# Effects of *Curcuma longa* extract on cognitive function in healthy participants with subtle cognitive weaknesses within the normal range: a randomized, double-blind, placebo-controlled trial

**DOI:** 10.3389/fnut.2026.1866285

**Published:** 2026-07-30

**Authors:** Ryosuke Saji, Ryusei Uchio, Kazuki Asada, Chinatsu Okuda-Hanafusa, Ami Tokaji, Koutarou Muroyama, Shinji Murosaki, Yoshihiro Yamamoto, Yoshitaka Hirose, Kengo Kawasaki

**Affiliations:** Research & Development Institute, House Wellness Foods Corp., Itami, Hyogo, Japan

**Keywords:** anti-inflammation, attention, bisacurone, cognitive function, *Curcuma longa* (turmeric), neuroinflammation, orientation, turmeronol

## Abstract

**Introduction:**

The dietary spice Curcuma longa *(C. longa)*, also known as turmeric, has various physiological effects. A mixture of hot water extract and supercritical carbon dioxide extract of *C. longa* (CLE) containing the anti-inflammatory compounds turmeronols A and B and bisacurone has demonstrated anti-inflammatory activity. Although neuroinflammation is known to be associated with cognitive impairment, the effects of CLE on cognitive function remain unclear. In the present study, we investigated the effects of CLE and of its active ingredients, turmeronols A and B and bisacurone, on cognitive function.

**Methods:**

We conducted a randomized, double-blind, placebo-controlled trial in healthy participants aged 40 to 69 years with subtle cognitive weaknesses within the normal range. Participants were allocated to study groups based on a randomization schedule and took two capsules containing CLE (CLE group, *n* = 20) or two placebo capsules (placebo group, *n* = 20) daily for 24 weeks. Cognitive functions were evaluated at weeks 0, 12, and 24 using various cognitive assessments.

**Results:**

Among 35 cognitive endpoints, the CLE group showed significantly higher orientation scores at 24 weeks than the placebo group, as measured by the eye-tracking cognitive assessment and the Japanese version of the Montreal Cognitive Assessment. In addition, the CLE group showed significantly higher attention scores at 24 weeks than the placebo group, as measured by the eye-tracking cognitive assessment.

**Conclusion:**

These results suggest the possibility that CLE might improve the cognitive functions associated with orientation and attention.

**Trial Registration:**

UMIN-CTR, UMIN000054532. Registered 31 May 2024, https://center6.umin.ac.jp/cgi-open-bin/ctr/ctr_view.cgi?recptno=R000062302.

## 1 Introduction

Cognitive function encompasses multiple processes that influence an individual's ability to learn, remember, judge, decide, and plan (1, 2). It is typically assessed across several domains, including verbal and visual memory, attention, executive functions, and orientation. These functions decline with aging and in the presence of various pathological factors, including inflammation (3, 4). Age-related cognitive decline may progress through an intermediate stage of mild cognitive impairment (MCI) and ultimately develop into dementia, most commonly Alzheimer's disease (5, 6).

As the global population ages, preserving and improving cognitive function to prevent dementia has become a public health priority (7). Dementia is a leading cause of long-term care among older adults, and its progression markedly reduces quality of life (QOL) not only for affected individuals but also for their families (8, 9), thereby increasing social and economic burdens. Despite extensive research, no established methods exist to reliably prevent cognitive decline, and no effective treatments for dementia exist. Moreover, cognitive function has been reported to begin declining gradually from the late twenties (10, 11), highlighting the importance of early intervention to maintain cognitive abilities.

Inflammation is a biological response of the immune system to infection or tissue injury, typically involving acute and chronic phases. Chronic low-grade inflammation has been implicated in a range of pathologies, including cognitive decline and dementia (12–16). Inflammatory processes have been observed in the early stages of Alzheimer's disease, prior to the onset of dementia (17), and individuals with acute or chronic inflammation exhibit a 2–4-fold higher rate of cognitive decline than those without inflammation (18).

The central nervous system can also mount inflammatory responses, known as neuroinflammation, which are exacerbated by aging and lifestyle factors, such as an unbalanced diet, physical inactivity, and psychosocial stress (19–23). Neuroinflammation is implicated in the pathogenesis of psychiatric and neurodegenerative disorders, including Alzheimer's disease, amyotrophic lateral sclerosis, multiple sclerosis, and Parkinson's disease (24, 25). Microglia, the resident immune cells of the central nervous system, play a crucial role in maintaining neural homeostasis (26, 27). Upon activation, microglia produce inflammatory mediators such as interleukin (IL)-1β, IL-6, and tumor necrosis factor α (TNF-α). Excessive or chronic activation of microglia leads to sustained release of these cytokines, contributing to neuroinflammation (28–31). Although transient activation can be protective, prolonged activation disrupts synaptic plasticity and neurogenesis, ultimately impairing cognitive function (32, 33). This neuroinflammatory response is therefore closely linked to the development of cognitive impairment and neurodegenerative diseases (34–36).

Recent studies suggest that consuming foods with a diverse range of bioactive compounds may help mitigate age-related cognitive decline (37–39). International consensus reports indicate that dementia may be partially preventable, with up to 40%−45% of cases theoretically avoidable through modification of risk factors such as physical activity, diet, smoking, and obesity (3, 4). Among preventive strategies, dietary and nutritional interventions have attracted considerable attention as practical approaches. Improvements in diet quality may contribute to prevention of dementia by reducing associated risk factors (40). In fact, food-derived bioactive components with antioxidant and anti-inflammatory properties have shown potential to improve cognitive function (41–44). Higher curry consumption was reported to be associated with better cognitive function and decreased risk of MCI and dementia in middle-aged and older adults (45, 46).

Turmeric (*Curcuma longa* (*C. longa*)), a member of the Zingiberaceae family, is a key ingredient in curry and is widely consumed in many Asian diets. Curcumin, a lipophilic polyphenol and major component of *C. longa*, exerts a range of physiological effects (47), including anti-oxidant and anti-inflammatory effects (48, 49). In addition, curcumin-free *C. longa* water extracts have been reported to exhibit diverse biological activities (50). Other constituents of *C. longa* include the sesquiterpenoids turmeronols A and B and bisacurone (51, 52). Turmeronols inhibit the production of inflammatory mediators in microglial cells by suppressing the nuclear factor kappa-light-chain-enhancer of activated B cells (NF-kB) signaling pathway (53), and bisacurone inhibits monocyte adhesion to endothelial cells through downregulation of vascular cell adhesion molecule 1 (VCAM-1) expression and NF-kB signaling (54). *C. longa* water extracts have been reported to exert anti-inflammatory effects (52, 55–57), prevent skeletal muscle atrophy (58), reduce fatigue (59), increase dermal water content (60), and improve depressive symptoms (61). Supercritical carbon dioxide extracts of *C. longa* have also been shown to suppress carrageenan-induced inflammation (62). Furthermore, daily intake of a mixture of hot water extract and supercritical carbon dioxide extract of *C. longa* (CLE) has demonstrated anti-inflammatory effects and improvements in insulin resistance and mental health–related QOL scores (56, 63, 64). However, the effects of CLE on cognitive function remain unclear.

The aim of this study was to evaluate the effects of CLE on cognitive function in healthy middle-aged and older adults with subtle cognitive weaknesses within the normal range, using a randomized, double-blind, placebo-controlled trial.

## 2 Materials and methods

### 2.1 Study design

We conducted a 24-week, randomized, double-blind, placebo-controlled study from May 2024 to January 2025 at the Takara Clinic in Tokyo, Japan, using a contract research organization (CRO; ORTHOMEDICO Inc, Tokyo, Japan). The study was registered with the University Hospital Medical Information Network (UMIN; registration number, UMIN000054532; registered May 31, 2024; https://center6.umin.ac.jp/cgi-open-bin/ctr/ctr_view.cgi?recptno=R000062302), approved by the Ethics Committee of Takara Clinic (Approval ID: 2405-00385-0050-10-TC; May 9, 2024), and performed in accordance with the principles of the Declaration of Helsinki (2013). All participants provided written informed consent. The study was designed and conducted in accordance with the Consolidated Standards of Reporting Trials (CONSORT) 2010 statement (65). A completed CONSORT checklist based on the 2025 update is provided in Supplementary Table S1.

### 2.2 Participant enrollment

Participants were recruited from May to June 2024 by ORTHOMEDICO Inc. A total of 78 individuals visiting the Takara Clinic were consecutively assessed for eligibility. Inclusion criteria were as follows: (1) healthy Japanese men and women aged 40 −69 years, (2) a Japanese version of the Mini-Mental State Examination (MMSE-J) score ≥ 24 (66) and a Japanese version of the Montreal Cognitive Assessment (MoCA-J) score ≥ 20 (67) at screening to exclude individuals with dementia, (3) validity indicators of the Cognitrax (Health Solution, Inc., Tokyo, Japan) verbal and visual memory tests both rated as “yes” at screening, and (4) relatively low standardized Cognitrax composite memory scores at screening. Exclusion criteria were as follows: (1) current or past severe disease affecting the heart, liver, kidneys, or gastrointestinal tract; (2) current or past cardiovascular disease, including malignant tumors, heart failure, or myocardial infarction; (3) presence of a pacemaker or implantable cardioverter-defibrillator; (4) current treatment for chronic conditions such as arrhythmia, liver dysfunction, chronic kidney disease, cerebrovascular disease, rheumatic disease, diabetes mellitus, dyslipidemia, or hypertension; (5) diagnosis of dementia; (6) suspected insomnia; (7) current or past psychiatric disorder (including depressive symptoms); (8) daily alcohol intake of ≥ 40 g for men or ≥ 20 g for women; (9) smoking; (10) impaired function of both hands; (11) extremely irregular eating or lifestyle habits (e.g., shift work or night shifts); (12) consumption of nutraceutical or functional foods with health claims; (13) use of pharmaceuticals (including Kampo medicines) or supplements, including psychotropic drugs (e.g., antipsychotics, anxiolytics, antidepressants, antiparkinsonian agents, antiepileptics) or anticoagulants; (14) drug or food allergies (including turmeric allergy); (15) pregnancy, breastfeeding, or planned pregnancy during the study; (16) participation in another clinical trial within 28 days before consent or planned participation during the study; (17) blood donation exceeding the following limits: any donation within 1 month before consent, donation of 400 ml of whole blood within 3 months (men) or 4 months (women) before consent, or total donations of 1,200 ml (men) or 800 ml (women) within the past year; and (18) any other condition deemed unsuitable by the investigators.

### 2.3 Selection, randomization, and blinding

Of the 78 screened individuals, 40 participants who met the inclusion and exclusion criteria were selected by the CRO. The CRO test food dispatcher confirmed the indistinguishability of the test foods, verified the screening data, and assigned identification numbers to participants. An independent allocation manager, not otherwise involved in the study, generated a randomization schedule using R software (version 4.4.0) to randomly allocate participants to either the CLE or placebo group. The algorithm used block randomization with randomly varying block sizes and a 1:1 allocation ratio. Randomization was stratified by Cognitrax composite memory domain score, sex, and age at screening (placebo group, *n* = 20; CLE group, *n* = 20). The CRO test food dispatcher, who received the allocation table with coded study foods, was solely responsible for distributing the foods according to the allocation schedule. The authors randomly assigned numbers to the CLE and placebo capsules, and the CRO did the same for the participants; these randomization lists were kept in a secure location until unblinding. All participants and study investigators remained blinded to group allocation throughout the study.

### 2.4 Experimental foods

The composition of the capsules is shown in Table 1. The base capsules were composed of gelatin, glycerin, a soybean-derived emulsifier, and beeswax. Placebo capsules contained carob and tartrazine as coloring agents to match the CLE capsules. The CLE capsules contained a mixture of hot water extract and supercritical carbon dioxide extract of *C. longa* (Turmeric Extract Mixture, House Wellness Foods) containing turmeronols A and B and bisacurone. The ethics committee confirmed that the two capsule types were indistinguishable in appearance. *C. longa* extracts were prepared as previously described (56, 64, 68). In brief, the hot water extract was obtained by extracting rhizomes at 98 °C for 1 h, followed by concentration, mixing with dextrin, and spray drying. The supercritical carbon dioxide extract was obtained by extracting rhizomes at 65 °C and 150 bar for 6 h, followed by concentration, mixing with vegetable oil, and filtration through a 1-μm membrane. The two extracts were then combined to produce CLE.

**Table 1:** Composition of the test capsules.

Component	Placebo (0.96 g/2 capsules)	CLE (0.96 g/2 capsules)
Energy, Kcal	6.6	5.6
Carbohydrate, g	0.10	0.25
Protein, g	0.23	0.24
Lipid, g	0.59	0.40
Sodium chloride, mg	1.7	1.9
Bisacurone, μg	0	400
Turmeronol A, μg	0	100
Turmeronol B, μg	0	100

CLE, *Curcuma longa* extracts.

### 2.5 Intervention

The intervention period spanned July 2024 to January 2025. Participants ingested two CLE or placebo capsules per day for 24 weeks. Study visits were conducted at weeks 0, 12, and 24 and included a physician interview, physical assessments, and a cognitive evaluation. The primary outcome was the Cognitrax (CNS Vital Signs) composite memory score. The secondary outcomes were the other cognitive domains of Cognitrax, the MoCA-J, and the eye-tracking cognitive assessment. Hematology, biochemistry, and urinalysis tests were performed at each visit by a contracted laboratory (LSI Medience Corporation, Tokyo, Japan). Participants were permitted to drink only water for 6 h before blood sampling. During the study, participants recorded capsule intake, medication use, and (for women) menstruation in daily diaries. Participants were instructed to comply with the following rules throughout the study: (1) take the study capsules according to the specified dosage and administration instructions with ≥ 90% adherence; (2) maintain usual lifestyle habits, including diet, exercise, and alcohol use; (3) avoid consuming nutraceutical or functional foods (excluding study capsules); and (4) avoid excessive alcohol consumption, extreme exercise, fasting, and unbalanced diets. The safety of CLE was confirmed in a previous clinical study at doses up to 5 times as high as in this study, with no adverse effects observed on physical measurements, hematology, biochemistry, or urinalysis (64).

### 2.6 MMSE-J

The MMSE-J is the Japanese version of the Mini-Mental State Examination (69), a brief face-to-face screening tool for dementia (70, 71) with high reliability and validity (72, 73). Scores range from 0 to 30, with higher scores indicating better cognitive function and a cut-off of 23/24 indicating possible dementia (74–76). In this study, the MMSE-J was used to confirm the absence of dementia (score ≥ 24).

### 2.7 Cognitrax tests

Cognitrax (CNS Vital Signs) is a computerized neurocognitive test battery with established reliability and validity (77–80). It includes 10 tests measuring reaction time and accuracy. Results were standardized (mean 100, standard deviation (SD) 15) based on age-matched norms, with higher scores indicating better cognitive function.

### 2.8 MoCA-J

The MoCA-J is the Japanese version of the Montreal Cognitive Assessment (67), a brief face-to-face screening tool for MCI (81, 82) with high reliability and validity (83, 84). It includes the following 12 subtests: alternating trail making, visuoconstruction skills (cube and clock), naming, digit span, vigilance, serial sevens, sentence repetition, verbal fluency, abstraction, delayed recall, and orientation. The total score ranges from 0 to 30, with higher scores indicating better cognitive function and a cut-off of 25/26 indicating possible MCI (67, 85, 86). Previous studies have shown that anti-inflammatory drugs and functional foods improve MoCA scores (87, 88).

### 2.9 Eye-tracking cognitive assessment

An eye-tracking–based cognitive assessment application (Ai-BrainScience, Inc., Osaka, Japan) was used to evaluate domains including memory, attention, visuospatial function, language, and orientation (89, 90). The system uses facial recognition and a tablet-based camera (iPad Pro, iPadOS) to track gaze. Cognitive scores were calculated based on fixation time on correct response areas during task-based video stimuli. Higher scores indicate better cognitive function. Standardized calibration and preprocessing procedures were used. Detailed descriptions of the video stimuli and scoring methodology have been reported previously (90). The system has demonstrated equivalence to approved software as a medical device (SaMD) in Japan (approval no. 30500BZX00235000) and has high reliability and validity (90).

### 2.10 Sample size

Sample size was calculated using G^*^Power 3.1.9 (University of Düsseldorf, Germany) based on a previous study showing 132% improvement in Cognitrax composite memory with astaxanthin (91), which has anti-inflammatory effects (92, 93). Assuming 132% improvement in Cognitrax composite memory by CLE, Cohen's d of 0.96, statistical power of 80%, and type 1 error of 5%, at least 18 participants per group were required. To account for a 10% dropout rate, 20 participants per group were enrolled.

### 2.11 Statistical analysis

The intention-to-treat (ITT) population, comprising all randomized participants, was used for efficacy analyses. No protocol deviations occurred. Analyses were performed using IBM SPSS Statistics version 26 for Windows (IBM Corp., Armonk, NY, USA). Results are presented as mean and SD. Between-group comparisons at baseline were performed using Welch's *t*-test. Within-group changes were assessed using paired *t*-tests. Between-group differences in cognitive outcomes were analyzed using a mixed-effects repeated measures model (94, 95), with baseline values included as a covariate. Missing outcome data were handled without imputation. The primary statistical inference was based on the group × time interaction, with statistical significance set at *p* < 0.05.

Safety was evaluated in the safety analysis population (SAF), defined as all participants except those who (1) did not receive the allocated intervention or (2) did not undergo a safety assessment. Adverse events and reactions were collected through participant reports (interviews at each assessment point) and clinical evaluations by study physicians, and incidence rates were calculated for each group. For each participant, abnormal findings in urinalysis and blood tests after the intervention were recorded, and the proportion of participants with abnormal findings was calculated by group. Incidence rates were compared between groups using the chi-square test. In addition, study physicians reviewed all safety data, including physical examinations, laboratory results, and reported symptoms, to assess the causal relationship between adverse events and test food intake.

## 3 Results

### 3.1 Participant characteristics

**Figure 1:**
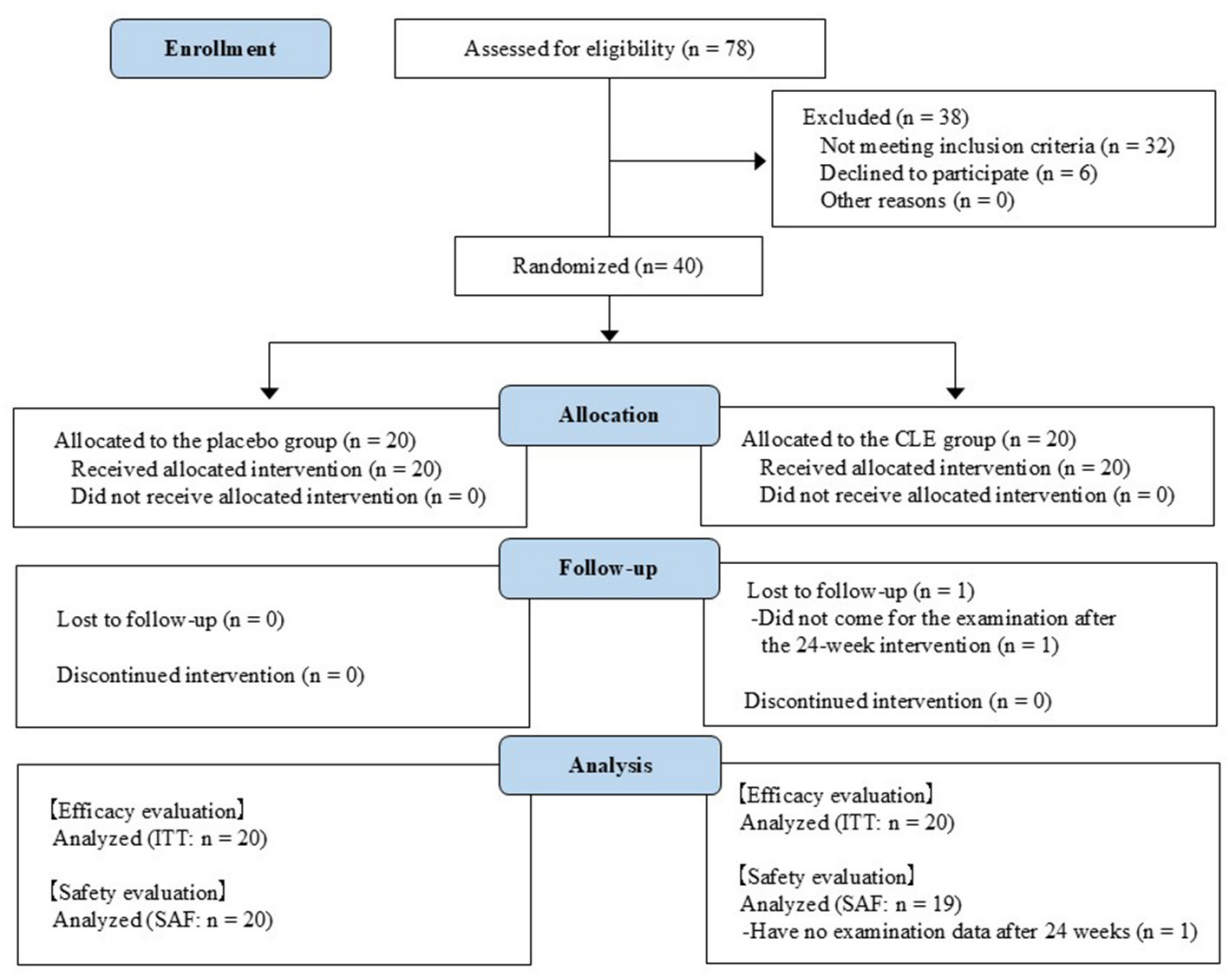
Study flow diagram (CONSORT 2025). CLE, *Curcuma longa* extracts; CONSORT, Consolidated Standards of Reporting Trials; ITT, intention-to-treat; SAF, safety analysis population.

The study flow diagram is shown in Figure 1. Out of 78 potential participants, 40 were randomly allocated to the CLE or placebo group (*n* = 20 per group). All participants received the allocated intervention; however, one participant in the CLE group did not participate in the examination at week 24. For the efficacy analysis, the relevant data were treated as missing values and analyzed (ITT population: *n* = 20 in the placebo group, *n* = 20 in the CLE group), whereas for the safety analysis, the relevant data were excluded from the analysis (SAF population: *n* = 20 in the placebo group, *n* = 19 in the CLE group). Baseline characteristics were not significantly different between groups (Table 2). Mean capsule intake did not differ significantly between groups (data not shown).

**Table 2:** Baseline characteristics of the participants^a^.

Characteristics	Placebo group (*n* = 20)	CLE group (*n* = 20)
Sex, male/female, *n*	10/10	10/10
Age, years	52.7 ± 6.8	53.4 ± 7.5
Education, years	13.9 ± 2.2	14.2 ± 2.0
Physical measurements and tests		
BMI, kg/m^2^	22.1 ± 4.0	22.5 ± 4.9
SBP, mmHg	111.6 ± 18.8	113.5 ± 15.4
DBP, mmHg	71.1 ± 12.7	73.5 ± 13.0
Cognitive function		
MMSE-J total score	28.6 ± 1.3	28.7 ± 1.1
MoCA-J total score	25.1 ± 2.5	24.8 ± 1.7
Cognitrax composite memory score	79.3 ± 12.6	79.9 ± 13.1

CLE, *Curcuma longa* extracts; BMI, body mass index; SBP, systolic blood pressure; DBP, diastolic blood pressure; MMSE-J, Japanese version of Mini-Mental State Examination; MoCA-J, Japanese version of Montreal Cognitive Assessment.

^a^Values represent means ± standard deviations. Sex distribution in each group was compared with Welch's *t*-test. Cognitive functions were compared with two-tailed unpaired Student's *t*-tests when variance was homogeneous and Welch's *t*-test when variance was heterogeneous.

### 3.2 Effect of CLE on Cognitrax test scores

There were no significant differences (*p* = 0.0501) at 24 weeks in the Cognitrax reaction time score and its change from baseline between groups. Other Cognitrax scores also did not differ significantly between groups (Table 3).

**Table 3:** Effect of CLE on Cognitrax test scores ^a^.

Cognitive domains		Absolute values			Change from baseline	
		Before intake	Week 12	Week 24	Week 12	Week 24
Neurocognitive index	Placebo	99.1 ± 8.3	94.2 ± 22.9	96.3 ± 24.5	−4.8 ± 21.9	−2.6 ± 23.6
	CLE	98.9 ± 6.7	100.6 ± 8.5	103.6^#^ ± 8.1	1.8 ± 7.9	4.3 ± 7.3
Composite memory	Placebo	79.3 ± 12.6	87.5^#^ ± 22.5	90.4^#^ ± 23.5	8.3 ± 17.3	11.1 ± 18.5
	CLE	79.9 ± 13.1	91.5^#^ ± 16.6	96.2^##^ ± 21.5	12.4 ± 21.5	16.2 ± 20.9
Verbal memory	Placebo	77.0 ± 16.3	92.5^##^ ± 24.3	95.8^##^ ± 19.7	15.5 ± 16.4	18.8 ± 17.9
	CLE	81.1 ± 20.6	98.8^##^ ± 17.2	102.1^##^ ± 23.3	18.7 ± 23.9	22.0 ± 17.7
Visual memory	Placebo	90.1 ± 12.0	88.3 ± 14.3	89.5 ± 22.5	−1.8 ± 16.5	−0.6 ± 21.6
	CLE	86.8 ± 12.6	87.7 ± 15.8	92.0 ± 16.3	1.3 ± 21.1	4.2 ± 24.9
Psychomotor speed	Placebo	102.7 ± 11.6	104.7 ± 18.0	107.3^#^ ± 15.0	2.0 ± 13.0	4.6 ± 9.4
	CLE	104.2 ± 12.0	105.6 ± 9.7	107.8^#^ ± 12.6	1.7 ± 11.3	4.2 ± 8.6
Reaction time	Placebo	99.5 ± 10.3	96.2 ± 14.6	95.8 ± 12.8	−3.3 ± 12.6	−3.7 ± 10.9
	CLE	100.6 ± 12.5	102.1 ± 14.2	103.2 ± 9.4	0.9 ± 11.5	1.3 ± 8.2
Complex attention	Placebo	108.4 ± 10.2	86.2 ± 64.1	84.6 ± 95.9	−22.2 ± 62.7	−23.8 ± 96.9
	CLE	106.1 ± 14.7	101.6 ± 13.6	104.3 ± 11.3	−4.2 ± 15.7	−1.7 ± 10.3
Cognitive flexibility	Placebo	104.1 ± 9.9	96.5 ± 25.4	103.3 ± 13.9	−7.6 ± 24.5	−0.8 ± 15.6
	CLE	104.7 ± 11.8	103.2 ± 8.1	106.4 ± 10.7	−1.2 ± 10.5	1.5 ± 10.3
Processing speed	Placebo	111.0 ± 10.9	112.5 ± 16.2	115.0 ± 12.2	1.5 ± 14.3	4.0 ± 12.5
	CLE	110.5 ± 13.3	110.8 ± 14.3	115.3^#^ ± 14.2	0.8 ± 9.1	4.5 ± 8.7
Executive function	Placebo	104.1 ± 9.8	96.8 ± 25.0	103.1 ± 13.7	−7.4 ± 23.8	−1.0 ± 15.6
	CLE	104.6 ± 11.2	103.7 ± 8.4	107.0 ± 10.3	−0.5 ± 10.0	2.2 ± 10.5
Social acuity	Placebo	89.2 ± 20.2	86.8 ± 27.9	92.7 ± 21.2	−2.4 ± 28.2	3.6 ± 21.1
	CLE	85.0 ± 19.7	87.3 ± 22.2	98.3 ± 13.7	3.7 ± 30.5	11.9 ± 25.7
Non- verbal reasoning	Placebo	103.8 ± 14.9	100.6 ± 13.2	104.9 ± 12.4	−3.2 ± 19.0	1.2 ± 13.5
	CLE	98.9 ± 14.3	98.4 ± 15.8	99.7 ± 14.9	−1.0 ± 20.1	1.8 ± 18.0
Working memory	Placebo	103.1 ± 13.8	105.2 ± 11.9	106.7 ± 13.5	2.1 ± 11.3	3.6 ± 15.1
	CLE	102.2 ± 14.7	101.0 ± 16.4	104.1 ± 13.7	−0.8 ± 12.2	1.2 ± 13.0
Sustained attention	Placebo	100.3 ± 18.4	102.6 ± 24.0	103.3 ± 18.2	2.3 ± 12.2	3.1 ± 19.8
	CLE	105.5 ± 11.3	104.6 ± 12.1	108.2 ± 8.5	−0.5 ± 8.3	2.1 ± 8.9
Simple attention	Placebo	98.7 ± 12.7	45.3 ± 184.8	−2.6 ± 416.9	−53.4 ± 185.8	−101.3 ± 415.5
	CLE	99.4 ± 17.3	90.3 ± 36.4	96.7 ± 16.7	−8.7 ± 35.7	−3.5 ± 25.9
Motor speed	Placebo	96.2 ± 14.1	98.2 ± 16.1	100.1 ± 14.9	2.0 ± 11.1	4.0 ± 9.5
	CLE	98.8 ± 16.0	100.3 ± 11.8	100.4 ± 14.7	1.6 ± 13.4	2.6 ± 9.5

CLE, *Curcuma longa* extracts.

^a^Values represent means ± standard deviations at baseline, week 12, and week 24 for *n* = 20 (placebo group) or *n* = 20 (CLE group). Between-group differences were evaluated using a mixed-effects repeated measures model, and within-group differences from baseline were evaluated using paired *t*-tests (^#^*p* < 0.05, ^##^*p* < 0.01).

### 3.3 Effect of CLE on MoCA-J scores

At 24 weeks, the MoCA-J orientation score and its change from baseline were significantly higher in the CLE group than in the placebo group (*p* < 0.05; Table 4). Other MoCA-J scores did not differ significantly between the two groups.

**Table 4:** Effect of CLE on MoCA-J scores ^a^.

Cognitive domains		Absolute values			Change from baseline	
		Before intake	Week 12	Week 24	Week 12	Week 24
Total score	Placebo	25.1 ± 2.5	26.0 ± 3.0	26.0 ± 2.0	0.9 ± 2.1	0.9 ± 2.3
	CLE	24.8 ± 1.7	26.1^#^ ± 2.3	26.6^##^ ± 2.2	1.2 ± 2.4	1.9 ± 2.3
Subcategory						
Alternating trail making	Placebo	1.0 ± 0.2	1.0 ± 0.0	1.0 ± 0.0	0.1 ± 0.2	0.1 ± 0.2
	CLE	1.0 ± 0.2	0.9 ± 0.2	1.0 ± 0.0	0.0 ± 0.3	0.1 ± 0.2
Visuoconstruction skills (cube)	Placebo	0.5 ± 0.5	0.6 ± 0.5	0.7 ± 0.5	0.1 ± 0.4	0.2 ± 0.6
	CLE	0.7 ± 0.5	0.6 ± 0.5	0.7 ± 0.5	−0.1 ± 0.7	0.1 ± 0.4
Visuoconstruction skills (clock)	Placebo	2.6 ± 0.6	2.6 ± 0.6	2.5 ± 0.7	0.1 ± 0.7	−0.1 ± 0.6
	CLE	2.5 ± 0.6	2.7 ± 0.5	2.5 ± 0.6	0.2 ± 0.6	0.1 ± 0.8
Naming	Placebo	3.0 ± 0.2	3.0 ± 0.0	3.0 ± 0.2	0.1 ± 0.2	0.0 ± 0.3
	CLE	3.0 ± 0.0	3.0 ± 0.0	2.9 ± 0.2	0.0 ± 0.0	−0.1 ± 0.2
Digit span	Placebo	2.0 ± 0.0	2.0 ± 0.2	2.0 ± 0.0	−0.1 ± 0.2	0.0 ± 0.0
	CLE	1.9 ± 0.4	1.9 ± 0.3	1.7 ± 0.7	0.0 ± 0.3	−0.1 ± 0.3
Vigilance	Placebo	1.0 ± 0.0	1.0 ± 0.0	0.9 ± 0.3	0.0 ± 0.0	−0.1 ± 0.3
	CLE	1.0 ± 0.0	1.0 ± 0.0	1.0 ± 0.0	0.0 ± 0.0	0.0 ± 0.0
Serial sevens	Placebo	2.9 ± 0.5	2.8 ± 0.6	2.9 ± 0.4	−0.1 ± 0.7	0.0 ± 0.5
	CLE	3.0 ± 0.2	2.8 ± 0.5	2.7 ± 0.6	−0.2 ± 0.6	−0.2 ± 0.6
Sentence repetition	Placebo	1.1 ± 0.7	1.4 ± 0.7	1.2 ± 0.8	0.3 ± 0.8	0.2 ± 0.7
	CLE	1.0 ± 0.8	1.2 ± 0.9	1.5^##^ ± 0.6	0.2 ± 0.8	0.6 ± 0.8
Verbal fluency	Placebo	0.6 ± 0.5	0.6 ± 0.5	0.6 ± 0.5	0.0 ± 0.6	0.0 ± 0.6
	CLE	0.3 ± 0.4	0.4 ± 0.5	0.4 ± 0.5	0.2 ± 0.5	0.2 ± 0.6
Abstraction	Placebo	1.3 ± 0.8	1.6 ± 0.7	1.6 ± 0.6	0.3 ± 0.7	0.3 ± 1.0
	CLE	1.6 ± 0.6	1.8 ± 0.4	1.5 ± 0.6	0.3 ± 0.7	−0.1 ± 0.9
Delayed recall	Placebo	3.6 ± 1.5	4.0 ± 1.4	4.2 ± 1.2	0.4 ± 1.4	0.6 ± 1.4
	CLE	3.5 ± 1.4	4.1 ± 0.7	4.6^##^ ± 0.6	0.6 ± 1.5	1.2 ± 1.4
Orientation	Placebo	5.8 ± 0.4	5.7 ± 0.5	5.7 ± 0.5	−0.2 ± 0.4	−0.1 ± 0.6
	CLE	5.5 ± 0.6	5.6 ± 0.6	5.9^*, *##*^ ± 0.3	0.2 ± 0.8	0.4^*^± 0.5

CLE, *Curcuma longa* extracts; MoCA-J, Japanese version of Montreal Cognitive Assessment.

^a^Values represent means ± standard deviations at baseline, week 12, and week 24 for *n* = 20 (placebo group) or *n* = 20 (CLE group). Between-group differences were evaluated using a mixed-effects repeated measures model (^*^p < 0.05), and within-group differences from baseline were evaluated using paired *t*-tests (^#^*p* < 0.05, ^##^*p* < 0.01).

### 3.4 Effect of CLE on eye-tracking cognitive assessment scores

At 24 weeks, the eye-tracking cognitive assessment attention score and its change from baseline were significantly higher in the CLE group than in the placebo group (*p* < 0.05; Table 5). Similarly, the orientation score and its change from baseline were also significantly higher in the CLE group (*p* < 0.05; Table 5). Other eye-tracking cognitive assessment scores did not differ significantly between groups.

**Table 5:** Effect of CLE on eye-tracking cognitive assessment score ^a^.

Cognitive domains		Absolute values			Change from baseline	
		Before intake	Week 12	Week 24	Week 12	Week 24
Total score	Placebo	70.7 ± 9.7	66.7 ± 14.9	69.3 ± 14.6	−4.0 ± 16.3	−1.4 ± 17.8
	CLE	64.1 ± 14.4	68.6 ± 11.9	73.0 ± 16.0	3.9 ± 18.1	9.0 ± 25.0
Subcategory						
Memory	Placebo	63.0 ± 11.7	61.1 ± 16.7	63.0 ± 15.6	−1.9 ± 18.0	0.0 ± 12.6
	CLE	57.7 ± 12.4	65.7^#^ ± 12.9	65.9 ± 15.4	8.4 ± 15.7	8.3 ± 20.5
Attention	Placebo	76.2 ± 26.7	87.4 ± 18.7	82.4 ± 18.6	11.2 ± 31.6	6.2 ± 32.6
	CLE	85.5 ± 11.0	83.9 ± 14.9	93.9^*^± 4.3	−1.5 ± 12.1	8.3^*^± 12.1
Visuospatial function	Placebo	52.3 ± 26.5	43.9 ± 28.0	43.3 ± 29.8	−8.5 ± 36.8	−9.0 ± 33.8
	CLE	44.5 ± 33.5	37.7 ± 23.4	55.6 ± 29.3	−7.5 ± 35.4	11.5 ± 46.6
Language	Placebo	85.2 ± 26.7	72.5 ± 36.9	79.2 ± 26.5	−12.7 ± 40.1	−6.0 ± 39.7
	CLE	63.3 ± 40.4	82.8 ± 31.9	65.0 ± 44.7	17.2 ± 47.6	2.5 ± 68.5
Orientation	Placebo	81.4 ± 8.2	80.4 ± 12.1	81.4 ± 13.5	−1.0 ± 11.2	0.1 ± 15.1
	CLE	78.7 ± 11.8	82.3 ± 10.4	88.8^*^± 7.8	2.6 ± 17.1	10.3^*^± 14.7

CLE, *Curcuma longa* extracts.

^a^Values represent means ± standard deviations at baseline, week 12, and week 24 for *n* = 20 (placebo group) or *n* = 20 (CLE group). Between-group differences were evaluated using a mixed-effects repeated measures model (^*^p < 0.05), and within-group differences from baseline were evaluated using paired *t*-tests (^#^*p* < 0.05).

### 3.5 Safety analysis

Adverse events were assessed in the ITT population (placebo group, *n* = 20; CLE group, *n* = 20). No adverse events were reported in either group.

Safety parameters, including hematology, biochemistry, urinalysis, and physical assessments, were evaluated in the SAF population (placebo group, *n* = 20; CLE group, *n* = 19). No significant differences were observed between groups. In addition, the study physician reviewed all safety data and identified no medically relevant changes associated with the test food.

## 4 Discussion

We conducted a 24-week, randomized, double-blind, placebo-controlled trial to investigate the effects of a mixture of hot water extract and supercritical carbon dioxide extract of *C. longa* (CLE), containing turmeronol A, turmeronol B, and bisacurone as potential active compounds, on cognitive function in healthy middle-aged and older adults with subtle cognitive weaknesses within the normal range. We found that, compared with placebo, CLE supplementation resulted in significantly higher orientation and attention scores on the eye-tracking cognitive assessment and significantly higher orientation scores on the MoCA-J. These results suggest that daily intake of CLE might improve the cognitive functions of orientation and attention.

Orientation is a fundamental cognitive function required for recognizing temporal, spatial, and personal information and is essential for daily functioning (96, 97). According to the International Classification of Functioning, Disability and Health developed by the World Health Organization, orientation functions include awareness of time (e.g., current date), place (e.g., current one's location, such as one's immediate surroundings), and person (e.g., one's own name and identity). Orientation is closely associated with global cognitive function and is included in many neuropsychological assessments as a key clinical indicator (67, 69, 98–101). Impairments in orientation often appear as early symptoms of cognitive impairment and in progression from MCI to Alzheimer's disease and are considered a useful early diagnostic marker of these disorders (102, 103). In the present study, CLE intake significantly improved orientation scores in two independent assessments (MoCA-J and eye-tracking tests; Tables 4, 5), suggesting a potential role in improving orientation functions.

Attention is the ability to maintain focus on relevant stimuli while filtering out distractions and underlies many higher-order cognitive processes (104, 105). According to the clinical model of attention by Sohlberg and Mateer (106), attentional functions can be classified into components including focused, sustained, selective, alternating, and divided attention. Impairments in attention can negatively affect communication and daily functioning; they appear early in the course of dementia and worsen with disease progression (107). In this study, CLE intake significantly improved attention scores (Table 5), suggesting a potential benefit in enhancing attentional processes.

Because orientation and attention are among the cognitive domains affected early in dementia, improvements in these functions may have broader implications. Although this study did not directly assess clinical outcomes such as MCI or dementia incidence, the observed improvements raise the possibility that CLE may reduce risk for these disorders. Nevertheless, because the sample size was determined according to the primary outcome, the findings for orientation and attention should be considered exploratory because of the limited statistical power. Further longitudinal studies are warranted to evaluate its effects on clinical endpoints.

Cognitive function is closely linked to QOL, defined as an individual's perception of their position in life within a cultural, social, and environmental context (108). Health-related QOL can be evaluated by the self-reported SF-36 questionnaire, which measures physical and mental health and has demonstrated good reliability and validity (109). In previous clinical studies of middle-aged and older adults, CLE significantly improved scores on the mental health subscale of the SF-36 (64), and *C. longa* water extracts improved scores on several SF-36 subscales, including vitality, mental health, and general health, as well as the mental component summary score (63). Cognitive dysfunction is associated with worse health-related QOL in older adults (110, 111), and cognitive function is a predictor of QOL (112). In particular, MoCA orientation scores are positively correlated with SF-36 QOL scores in middle-aged and older adults (113), and attention has been positively correlated with QOL in older adults (114). Additionally, multidomain lifestyle interventions plus multimorbidity management were associated with an improvement in the MoCA orientation score of approximately 0.2 points, accompanied by a significant improvement in SF-36 QOL scores in older adults (115). It is possible that the improvement of cognitive function associated with orientation and attention from CLE intake may partly contribute to the improvement of health-related QOL in older adults.

The frontal lobes, particularly the prefrontal cortex, play a central role in cognitive processes such as attention (116–122). Recently, the prefrontal cortex has also been implicated in the function of orientation (123). Age-related atrophy is pronounced in these regions, which may contribute to declines in these functions. Previous interventions targeting frontal lobe function, including aerobic exercise and cognitive training, have been shown to improve attention and orientation (124–128). Given that CLE improved these domains in the present study (Tables 4, 5), it is possible that its effects are mediated in part through modulation of frontal lobe–dependent functions.

Neuroinflammation is a key contributor to cognitive decline and dementia (129, 130). In animal studies, anti-inflammatory drugs attenuated cognitive decline (131). Although clinical findings have been inconsistent, anti-inflammatory drugs may have the potential to reduce the risk of Alzheimer's disease (132, 133). Microglial activation plays a central role in neuroinflammation (129, 130) and can be quantified using translocator protein-positron emission tomography (134–136). Previous studies have demonstrated age-related increases in microglial activation across multiple brain regions (137) and increased microglial activation in the frontal lobe of patients with MCI, dementia, and cognitive impairments (17, 138–142). Microglial inhibitors have been shown to attenuate neuroinflammation in the frontal lobe and other brain regions and to improve cognitive function in animal models (143–146). These inhibitors have also been reported to ameliorate cognitive deficits in patients with neuroinflammation-associated schizophrenia (147–149). In a previous study, turmeronols A and B, potential active ingredients of CLE, inhibited lipopolysaccharide-induced activation of microglial cells (53). In line with this finding, *C. longa* water extracts, which also contain turmeronol A and B, attenuated microglial activation and cognitive impairment in a mouse model of Alzheimer's disease (150). Taken together, these findings suggest that CLE may reduce neuroinflammation through effects on microglial activation, potentially contributing to the cognitive improvements observed in this study. However, direct evidence in humans is lacking, and further studies using neuroimaging approaches such as translocator protein-positron emission tomography are needed to confirm this mechanism.

Inflammatory mediators such as IL-1β, IL-6, and TNF-α can contribute to cognitive impairment and are involved in the pathogenesis of neurodegenerative diseases (28–31, 151). In addition, inflammatory stimulation of endothelial cells increases the expression of adhesion molecules, including VCAM-1, intercellular cell adhesion molecule-1 (ICAM-1), and E-selectin, which promote the infiltration of peripheral leukocytes into the brain, thereby exacerbating neuroinflammation and contributing to cognitive impairment (152, 153). In fact, anti-inflammatory drugs have shown the potential to improve cognitive functions in *in vivo* and clinical studies (131, 132). Additionally, functional food ingredients which inhibited the production of inflammatory mediators in animal studies improved orientation and attention scores in intervention studies (154–157). Previous studies have shown that CLE, as well as its components turmeronols A and B and bisacurone, inhibit the production of pro-inflammatory mediators (IL-1β, IL-6, TNF-α, and nitric oxide) in RAW264.7 macrophages, and that turmeronols similarly suppress these mediators in BV-2 microglial cells (52, 53, 56). In addition, *C. longa* water extracts and bisacurone inhibit the expression of VCAM-1, ICAM-1, and E-selectin in TNF-α-stimulated human umbilical vascular endothelial cells (54, 55), suppress phosphorylation of inhibitor of nuclear factor kappa B (IkB) kinase (IKK) and IkB-α, and inhibit activation of NF-kB, which regulates the expression of inflammatory mediators and adhesion molecules (52–55). In animal models, *C. longa* water extracts inhibit NF-kB activation and suppress mRNA expression of IL-1β, IL-6, TNF-α, and inducible nitric oxide synthase. These anti-inflammatory effects are accompanied by improved cognitive performance in the Morris water maze test (149). Other studies have shown that dietary supplementation with *C. longa* extracts attenuates neuroinflammation associated with fatigue (158), depression (61), and cognitive impairment (159, 160). Collectively, these findings suggest that CLE may reduce neuroinflammation by inhibiting inflammatory signaling pathways and the expression of inflammatory mediators and adhesion molecules, thereby contributing to the cognitive improvements observed in this study. Although the mechanism remains unclear, previous *in vitro* and animal studies suggest anti-inflammatory properties, which could be explored in future research. In addition, given the multiple eligibility criteria used in this study, these findings may not generalize to the broader population of middle-aged and older adults.

## 5 Conclusion

In this 24-week, randomized, double-blind, placebo-controlled trial, CLE supplementation improved orientation and attention scores in healthy middle-aged and older adults with subtle cognitive weaknesses within the normal range. These findings suggest that daily intake of CLE may support cognitive functions related to orientation and attention, potentially through anti-neuroinflammatory mechanisms.

## Data Availability

The raw data supporting the conclusions of this article will be made available by the authors, without undue reservation.
